# Editorial: Advances in plant hormone research in the face of a changing environment

**DOI:** 10.3389/fpls.2023.1239758

**Published:** 2023-07-25

**Authors:** Maren Müller, Ruozhong Wang, Guzel Kudoyarova

**Affiliations:** ^1^ Department of Evolutionary Biology, Ecology and Environmental Sciences, Faculty of Biology, University of Barcelona, Barcelona, Spain; ^2^ Hunan Provincial Key Laboratory of Phytohormones and Growth Development, Hunan Agricultural University, Changsha, China; ^3^ Ufa Institute of Biology, Ufa Federal Research Centre, Russian Academy of Sciences, Ufa, Russia

**Keywords:** plant hormones, climate change, biotic stress, abiotic stress, hormone signaling, hormone interaction, plant adaptation, plant stress tolerance

The worrying effects of global climate change pose major challenges for the future. Global warming, the evolution of pathogenic threats, and extreme weather conditions have a great impact on the plant ecosystem. Intense and unpredictable climate events are likely to exacerbate the food crisis, particularly in terms of ensuring global food security. Thus, climate-resilient agriculture is crucial to securing future harvests, and scientists around the world are involved in developing solutions. In this context, plant hormones, also known as phytohormones, play a pivotal role as they not only regulate plant growth and development but also regulate the plant’s ability to react to environmental changes. Plant hormones include a wide variety of compounds, including auxins, cytokinins, gibberellins, abscisic acid, salicylic acid, ethylene, jasmonates, brassinosteroids, strigolactones, and some peptides. Although it is now well known that plant hormones act through complex networks, many gaps remain regarding their interactions and feedback loops that connect their signaling pathways. This Research Topic aims to collect original research, perspective, and hypothesis and theory articles on our knowledge of recent advances in plant hormone research under changing environmental stress conditions.

The perspective article by Wang et al. presents a novel view on the plant transcription factor Dof (DNA-binding with one finger) as a key regulatory hub of abscisic acid, jasmonates, salicylates, and redox signaling in responses to abiotic stresses.

The research articles have addressed a number of aspects of plant hormone regulation to enhance crop production. He et al. studied the hormonal and source–sink regulation on the grain yield of ratoon rice, a resource-efficient rice production system in which a second rice crop is produced from the stubble after the main crop has been harvested. The authors reported that cytokinins appear to be the major signals regulating the sprouting of regenerated buds. Moreover, depending on the nodal position, cytokinins seem to interact with either gibberellins or abscisic acid. The research article by Huang et al. focused on the role of *SMALL AUXIN-UP RNAs (SAURs)* in the aquatic crop *Euryale ferox*. *EuSAURs* appear to act as a molecular node between light and indole-3-acetic acid, thus modulating the accumulation of indole-3-acetic acid through interaction with PIN family proteins to promote ovary enlargement and to increase seed size. In the third research article in this Research Topic, Gao et al. studied the effect of silicon in controlling harmful disease gummosis in peaches through repressing ethylene biosynthesis. This disease, triggered by both biotic and abiotic stressors, involves excessive gum formation that significantly weakens the vitality of the tree, destroying its branches and fruit. Ethylene that increases significantly under stressful conditions promotes gummosis. However, exogenous silicon application restricts gummosis development by upregulating the expression of *PpSAMDC (Prunus persica S-ADENOSYLMETHIONINE DECARBOXYLASE)*, leading to increased accumulation of polyamines, which in turn inhibit ethylene synthesis, likely via the *PpACS1* and *PpACS2* (*1-AMINOCYCLOPROPANE-1-CARBOXYLIC ACID SYNTHASE 1* and *2*) gene downregulation.

Finally, the hypothesis and theory article of this Research Topic gives us an interesting and new perspective on the regulatory role of plant hormones under northern climate conditions. Global warming is one of the reasons why Subarctic and Arctic regions are gaining increasing interest for agricultural cultivation. However, due to milder winters, these ecosystems are increasingly susceptible to expanded pathogens and insects. In this context, Mithöfer et al. hypothesized that jasmonates, whose biosynthesis can be regulated by light, may have a promoting effect on jasmonate synthesis under Artic summer light conditions (24 h), which in turn may lead to increased plant stress tolerance.

Overall, these articles provide a clear picture of the ongoing dynamic research on plant hormones in the context of environmental stress conditions. New insights have been gained into the role of plant hormones in regulating abiotic and biotic stress responses, including interactions within and between plant hormonal pathways and stress signals. Moreover, given the need to increase food production due to population growth and ongoing climate change, some of these articles offer promising approaches. We hope that these compiled articles will also help generate new ideas that pave the way for future research.

## Author contributions

All authors listed have made a substantial, direct, and intellectual contribution to the work and approved it for publication.

